# Proximal bronchiolar adenoma with malignant transformation to invasive mucinous adenocarcinoma with 4 years follow-up: a case report and literature review

**DOI:** 10.3389/fonc.2025.1491339

**Published:** 2025-01-29

**Authors:** Yuan-Hui Yang, Ke Yin, Jia-Qi Xu, Xiao-Ying Xu, Jun-Lei Zhang, Ji-Xuan Liu, Xin-Zhi Feng, Xiao-Yan Lin

**Affiliations:** ^1^ Department of Pathology, Shandong Provincial Hospital, Shandong University, Jinan, Shandong, China; ^2^ Department of Pathology, Shandong Provincial Hospital Affiliated to Shandong First Medical University, Jinan, Shandong, China

**Keywords:** bronchiolar adenoma, invasive mucinous adenocarcinoma, KRAS, CDK6, malignant transformation

## Abstract

**Background:**

Bronchiolar adenoma (BA) is a rare benign tumor originating in the bronchial mucosal epithelium and occurring primarily in the periphery of the lung. The most prominent histopathological feature of BA is a double-layer bronchial epithelium containing continuous basal cell layers. However, due to the high mutation frequency of the driver gene, there is still debate as to whether BA has the potential for malignant transformation. In frozen sections, basal cells are difficult to identify under the microscope, which makes it difficult to distinguish from mucinous adenocarcinoma, especially when BA malignancies transform into invasive mucinous adenocarcinoma (IMA), which can only be distinguished by histomorphological criteria, greatly increasing the difficulty of diagnosis.

**Case summary:**

In this paper, we present a case study of a 59-year-old man whose chest computed tomography (CT) revealed a progressively enlarging, high-density nodule over a four-year period in the outer basal segment of the right lower lobe. Consequently, he underwent thoracoscopic wedge resection of the right lower lobe. The postoperative pathological diagnosis revealed BA with mucous gland structure formation combined with partial basal cell loss, raising the possibility of malignant transformation into IMA. Regular postoperative follow-up showed no recurrence or metastasis. Hybridization Capture-based next-generation sequencing (NGS) analysis detected driver gene mutations in *Kirsten Rat Sarcoma viral oncogene homolog* (*KRAS*) and *Cyclin-Dependent Kinases* (*CDK*) *6* in the case, thereby inferring the malignant transformation of BA into IMA.

**Conclusion:**

In this case, the detection of driver gene *KRAS* mutation and loss of continuity in the basal cell layer within the mucous glandular structures of the nodule suggests the malignant transformation of BA into IMA, inferring the malignant potential of BA.

## Introduction

Ciliated muconodular papillary tumor (CMPT) is a rare pulmonary tumor originating from the peripheral bronchial epithelium, characterized by papillary structures and extracellular mucin. It consists of a dual-layered epithelial structure composed of ciliated cells, mucous cells, and continuous basal cells. Therefore, this non-dysplastic and non-invasive nodular growth tumor is named CMPT (1). However, in 2018, Chang et al. (2) reported cases lacking papillary structures, cilia, and mucous cells. Nevertheless, these lesions had bilayered cell structures containing continuous basal cell layers; as such, they used bronchiolar adenoma (BA) as a broader term for their lesions. Based on histomorphological and immunohistochemical findings, two types of BA have been identified: proximal-type, containing ciliated and mucous cells with papillary to flat architectural patterns, and distal-type, containing clara cells and type II pneumocytes, with a flat pattern (2–4). Complete diagnosis depends on differential diagnosis from mucinous adenocarcinoma (5, 6), particularly in frozen section diagnosis during surgery. Recently, there has been an extensive discussion on whether BA has malignant potential, and it has been concluded that the risk of malignant transformation is minimal. The Fifth Edition WHO for thoracic tumors classified it as a benign tumor, with an ICD-O code of 8140/0. In this paper, we present a rare case of malignant transformation of BA to invasive mucinous adenocarcinoma (IMA). Genetic analysis of the *KRAS* and *CDK6* driver genes revealed the same mutations in the BA region and IMA region of this lesion. The patient was followed-up for six months without any recurrence.

## Case presentation

**Figure 1:**
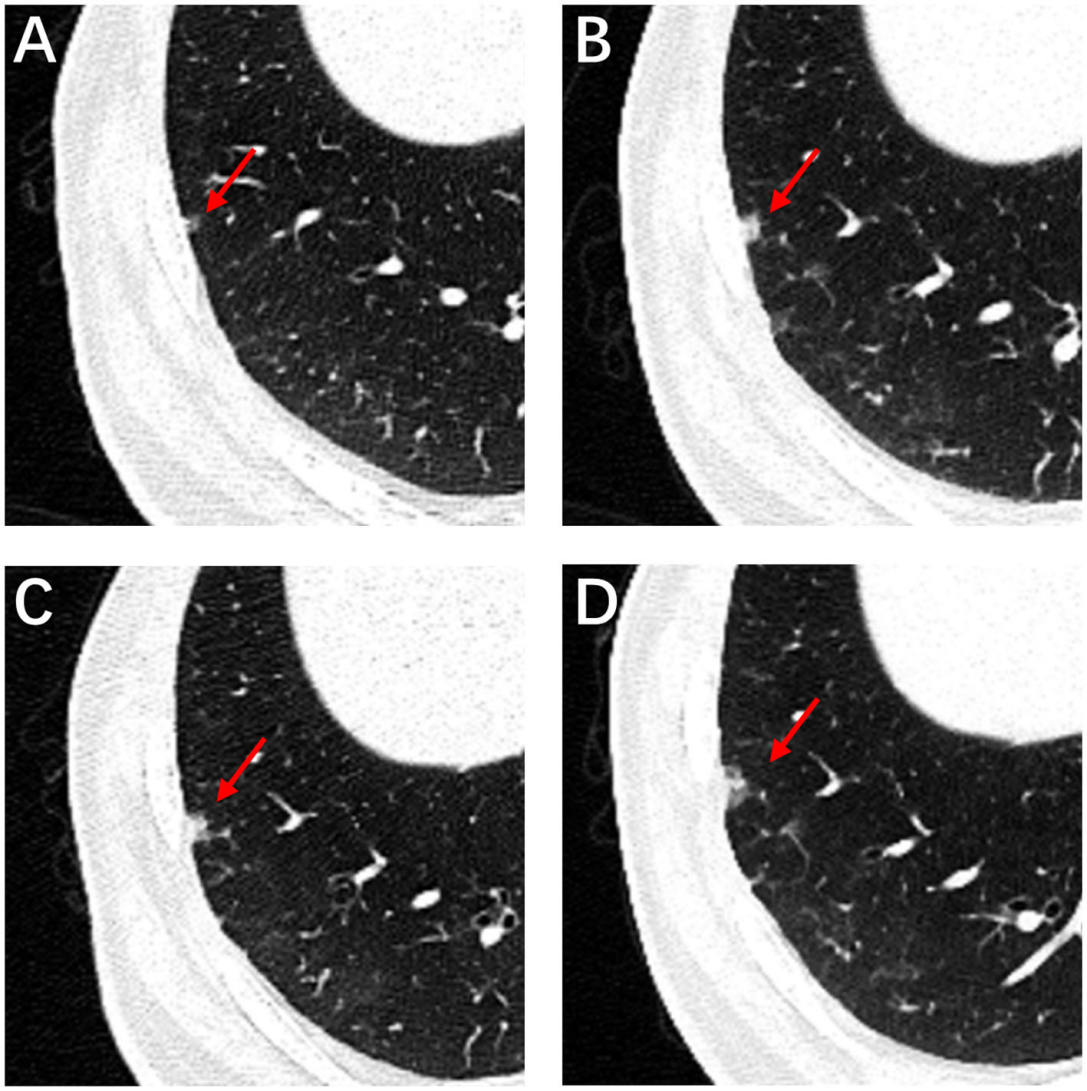
High-resolution computed tomography. CT image of the patient with subsolid nodule changes during 4 years. Initial CT image shows a subsolid nodule in the right lower lobe subpleural areas measured approximately 4 mm, with a round shape, smooth margin and clear tumor-lung interface (**A**, red arrow). CT imaging data from physical examination from 2018 to 2020 (**B–D**, red arrow).

**Figure 2:**
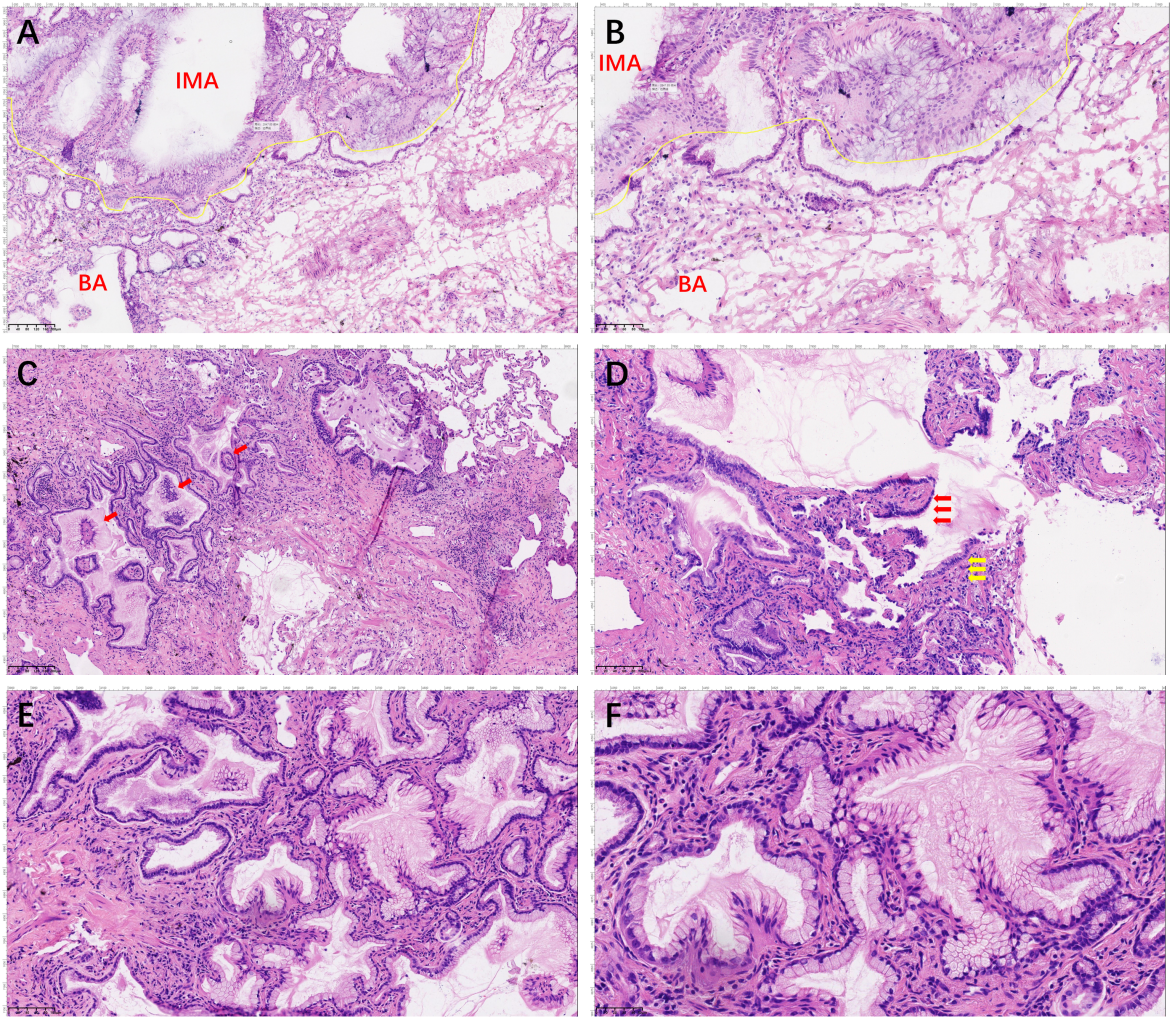
Hematoxylin and eosin staining of the whole tumor. The section shows the double-layered epithelial structure was seen to continue into tall columnar mucinous epithelium (**A**, 100× and **B**, 200×). The boundary of the nodule was unclear, with the papillary structure showing a skip growth pattern (red arrows; **C**, 200×). A continuous basal cell layer and a bilayered structure composed of ciliated cells (red arrow) or mucous cells (yellow arrow) growing along the alveolar wall (**D**, 200×). Tall columnar mucous cells lack ciliary structures and basal cell layers, with an increased nucleoplasmic ratio (**E**, 100× and **F**, 200×).

**Figure 3:**
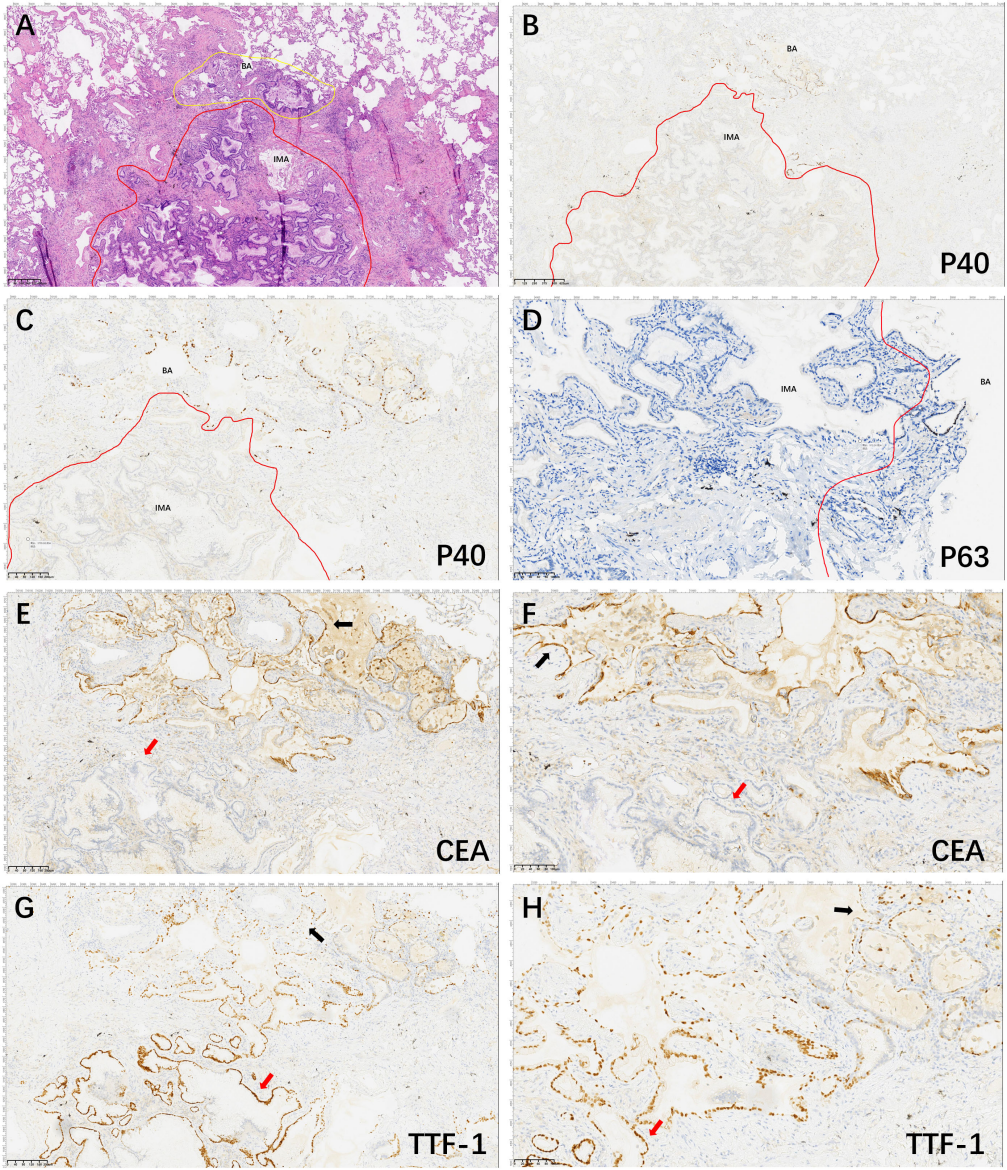
Hematoxylin and eosin and Immunohistochemical staining of the lesion area. The tumor consisted of two general areas: The area of BA (yellow line) and the area of IMA (red line) (**A**, 40×); P40 positive expression in basal cells in BA structures and basal cells in malignant IMA with absent expression (**B**, 40× and **C**, 100×). P63 positive expression in basal cells in BA structures and basal cells in malignant IMA with absent expression (**D**, 200×). CEA is continuously strongly and positively expressed in the BA structures (black arrow) and de-expressed in IMA structures (red arrow) (**E**, 100× and **F**, 200×). TTF1 is partially punctiformly expressed in BA structures (black arrow) and strongly positively expressed in IMA structures (red arrow) (**G**, 100× and **H**, 200×).

A 59-year-old man underwent thoracoscopic wedge resection of the right lower lobe on September 14, 2021. This procedure was conducted due to the presence of nodular hyperdense lesions in the subpleural region of the outer basal segment of the right lower lobe. The patient had undergone several chest CT scans over a period of four years from 2017, during which the nodule increased from 0.53 cm to 1.1 cm. The CT scans showed the gradual appearance of a marginal burr sign and central solid component over time (**Figures 1A–D**, red arrow). The patient’s serum tumor markers, including carcinoembryonic antigen (CEA), cancer antigens 125 (CA125), cytokeratin 19-fragments (CYFRA21-1), and squamous cell carcinoma antigen (SCC), were all within normal limits except for neuron specific enolase (NSE), which was observed to be elevated to a level of 30ng/ml. During intraoperative gross examination, a gray-white nodule, with a maximum cross-sectional area of 0.8 x 0.5 cm, was observed immediately adjacent to the pleura. Preliminary diagnosis based on frozen sections was bronchioloalveolar carcinoma (BA). However, microscopic observation revealed the presence of a bilayered cell structure transitioning into tall columnar mucinous epithelium (**Figures 2A, B**), indicating the possibility of well-differentiated mucinous adenocarcinoma. A paraffin section of postoperative hematoxylin-eosin staining showed an unclear tumor boundary and a papillary structure with a skip growth pattern (**Figure 2C**). Within this nodule, extracellular mucus with a continuous basal cell layer, as well as a bilayered structure composed of ciliated cells or mucous cells growing along the alveolar wall, were observed (**Figure 2D**). However, some glands were lined with proliferative mucinous cells, with an area of 0.4 x 0.3cm2. Tall columnar mucous cells lacked cilia structures and were slightly atypical, with enlarged nuclei, an increased ratio of nucleus to cytoplasm, not easily visible mitotic figures and loss of basal cell layer (**Figures 2E, F**). Hence, immunohistochemistry was performed with a panel including CK7, TTF-1, Napsin A, CK5/6, p63, p40, CEA and Ki-67. Immunohistochemical expression showed that P40 and P63 markers were continuously expressed in the part of BA, whereas the positive expression of P40 and P63 was lost in the mucoid glandular structure (**Figures 3A–D**). The expression pattern of CEA was similar to that of P40 and P63 (**Figures 3E, F**). TTF-1 was positively expressed in type II alveolar epithelium and clara cells. In this case, TTF1 was expressed punctiformly in the glands of BA, but continuously and strongly positively in glands formed by highly columnar mucous cells (**Figures 3G, H**). As a result of these findings, the final diagnosis was determined to be BA, with malignant transformation into well-differentiated mucinous adenocarcinoma considered in some areas.

To further investigate the molecular mechanisms present in BA and IMA lesions, as well as the potential correlation between them, NGS sequencing was utilized. Specifically, a panel covering 116 genes was used for analysis of the regions of BA and IMA.

All tissue collections and experiments were reviewed and approved by the Biomedical Research Ethic Committee of Shandong Provincial Hospital (NO. SWYX2024-542). The study was conducted in accordance with recognized ethical guidelines (Declaration of Helsinki). Informed consent was obtained from all of the participants.

The sample type tested in our experiment was the FFPE sample. Before performing NGS, the paraffin section of postoperative hematoxylin-eosin staining was used for annotation of the IMA region and BA region. The tumor cell content in each area both reached 70%. Corresponding areas in 10 slides of 5 μm thick paraffin sections were scraped with a knife to obtain IMA and BA tissues, respectively. DNA and RNA were extracted from IMA and BA tissues using the FFPE DNA/RNA kit (Amoy Diagnostics Co., Ltd. Xiamen). The library construction was conducted with the Human Cancer Multigene Mutation Detection kit (8.06.0056, Amoy Diagnostics Co., Ltd. Xiamen). This kit contains RNA gene fusion and DNA gene mutation detection systems. A total of 50ng RNA were used for reverse transcription with MiniAmpTM Thermal Cycler (Thermo Fisher Scientific Inc). A total of 100ng DNA/cDNA were used for hybridization with probes to specifically capture the target region. The captured fragments were enriched by extension, ligation, enzyme digestion, amplification and magnetic bead purification by Agencourt AMPure XP Kit (Beckman Coulter) to obtain the sequenced library. Quality control of sequencing libraries was performed with the 2100 Bioanalyzer system with matching reagents (Agilent) and the QuantusTM Fluorometer series with matching reagents (Promega). The library was sequenced using ADx-SEQ200 Plus (Amoy Diagnostics Co., Ltd. Xiamen). After Sequencing was completed, data were analyzed with the Illumina Sequencing Analysis Viewer, which yielded a Q30 base ratio of 86%. The average effective sequencing depth of library DNA was 1616×, and the RNA-Control was 7148×. The threshold for hot spot mutations was set at ≥0.5% with an alteration depth of ≥4, while for non-hot spot mutations, the threshold was ≥3% with the same alteration depth requirement. The copy number alteration (CNA) threshold for DNA was set at ≥3.5 copies, and the microsatellite instability (MSI) ratio threshold was set at ≥15%. For RNA fusions, the threshold was ≥10 copies, and for MET exon 14 skipping, it was ≥40 copies.

Of significant note, *KRAS* mutations (p.G12V, c.35G > T, Exon2) were found in both portions, although the abundance of these mutations differed. These results suggest that exists a close association between driver gene mutations and the process of malignant transformation of BA into IMA. Additionally, a mutation in the cyclin-dependent kinases (CDK) 6 gene was identified in the lesions, a cell cycle gene with a mutation abundance of 51.45% in IMA and 49.01% in BA (refer to **Table 1**). Recognition of the role played by *CDK6* as a cyclin D binding partner that controls the G1 phase and drives the cell cycle into the S phase is essential. Abemaciclib and Palbociclib can be used as the first selective inhibitor of *CDK6* (7, 8).

**Table 1:** Results of NGS.

Lesion type	Chr	Start	End	Ref	Alt	Depth	Freq	Gene	BLE
BA	chr12	25398284	25398284	C	A	1520	3.22%	KRAS	NM_033360.4:exon2:c.35G>T:p.(G12V):p.(Gly12Val)
BA	chr7	92247469	92247469	T	A	1875	49.01%	CDK6	NM_001145306.1:exon7:c.751A>T:p.(R251W):p.(Arg251Trp)
IMA	chr12	25398284	25398284	C	A	1520	0.44%	KRAS	NM_033360.4:exon2:c.35G>T:p.(G12V):p.(Gly12Val)
IMA	chr7	92247469	92247469	T	A	1549	51.45%	CDK6	NM_001145306.1:exon7:c.751A>T:p.(R251W):p.(Arg251Trp)

## Discussion

Ciliated muconodular papillary tumor (CMPT) is a rare pulmonary tumor originating from the peripheral bronchial epithelium, characterized by papillary structures and extracellular mucin. It consists of a dual-layered epithelial structure composed of ciliated cells, mucous cells, and continuous basal cells. Therefore, this non-dysplastic and non-invasive nodular growth tumor is named CMPT (1). In 2018, Chang et al. (2) analyzed 25 cases of proliferative nodules that arise from bronchiolar epithelium, with only four cases meeting the CMPT description. They discovered 17 cases of morphologically identical distal respiratory bronchiolar epithelium comprising flat structures with loss of ciliated and mucinous cells, replaced by apical cuboidal cells and clara cells with cytoplasmic snouts structures. Despite this, double epithelial structures containing continuous basal cell layers formed both types of nodules, with micropapillary tufts generated by ciliated cells present in the alveolar space. As a result, they summarized two types of BA: proximal-type and distal-type using the BA concept. The primary objective of considering this classification was to aid in diagnosing rather than categorizing each tumor into a specific proximal or distal pattern. BA malignant transformation is rare, with only three reported cases (**Table 2**). This case report describes a 59-year-old patient whose BA transformed malignantly into IMA.

**Table 2:** Summarized previously reported cases and molecular findings of malignant transformation of BA in the literature.

No.	Author	Size	Final diagnosis	Main genetic alterations
1	Han (6)	1.5×1.4 cm	BA transforming to IMA	KRAS mutations(G12V)
2	Li (14)	the mixed (Ground Glass Nodule) GGN is about 7 mm	BA with atypical hyperplasia and cancerization of adenocarcinoma in local area.	CCNE1 gene mutation (Exon7, c.476A > G)
3	Chen (5)	a 14 mm ground-glass opacity (GGO)	mucinous adenocarcinoma caused by the cancerization of CMPT (proximal BA)	no high frequency mutation
4	present case	1.1×0.7 cm	BA malignant transformation to IMA	KRAS mutations (p.G12V, c.35G > T, Exon2) and CDK6 mutations

BA is characterized by pure ground-glass and subsolid lesions in CT scans, with ill-defined peripheral opacity attributed to inflammatory infiltrates; lobular/spiculated margins indicate the probability of malignant invasion (9).

Distinguishing BA/CMPT from IMA in the frozen section is challenging. Although the frozen section reveals an area with glandular structures, consisting of a single layer of tall columnar mucous cells that lack obvious cellular atypia, the limitations of the frozen section make it difficult to differentiate between BA and IMA. Microscopic histomorphological features and the presence of basal cells are the only way to confirm the diagnosis. Diagnosis for cases with partial malignant transformation of BA is even more challenging. However, Intraoperative direct immunohistochemistry using basal cell markers such as P40, P63, or CK5/6 (10) can aid in diagnosing BA with malignant transformation to IMA during the frozen section. Moreover, postoperative paraffin sections revealed that basal cell loss in IMA is an important differential factor between BA and IMA that can be identified using immunohistochemical methods to label basal cells, such as P40, P63, or CK5/6, or TTF-1 for cuboidal cells not expressed in the mucous tall columnar cells of IMA. Liu et al. (11) suggests that BA may have carcinogenic potential, based on their use of a histological and immunohistochemical combination to diagnose a patient with BA accompanied by IMA.

Shao J (12) conducted a study where they sequenced 422 genes in 25 different samples. The study identified commonly mutated driver genes for BA, which include *EGFR* (52%), *KRAS* (16%), *ERBB2* (8%), *BRAF* (12%), and *RET* fusion (4%), which were consistent with the BA driver gene mutations reported by Chang (2) and other studies. These mutations are found in high frequency in BA driver genes, and combined with a skip growth pattern and characteristic micropapillary structure, there has been recent debate about BA’s potential as a precursor lesion (5, 6, 13, 14). In this particular case, we report the presence of *KRAS* mutations (G12V) in both the BA and IMA regions. In Chang’s study (15), it was observed that *KRAS* mutations (76%) and specific gene fusions frequently characterized the IMA genome, with the G12V mutation being the second most common mutation (32%) after G12D (36%). Moreover, *KRAS* mutations have been reported in 24% of BA (6). As such, we speculate that in this case, certain parts of the lesion converted gradually from a BA to an IMA due to *KRAS* mutations, which was also observed on the imaging as an increasing density of nodule. Supporting the argument of BA as a precursor lesion, Han (6) found that sequencing analysis of the BA and IMA regions showed that the occurrence of *KRAS* mutation (G12V) in both regions infers that the IMA originates from BA, malignant transformation of BA to IMA. Li (14) reported a case of *CCNE1* (Exon7, c.476A > G) mutation in mixed GGN and pure GGN, with mutation abundances of 48.83% and 45.41%, respectively. Overexpression of *CCNE1* has been shown to be a poor prognostic indicator in lung cancer and to contribute to the growth and metastasis of the disease (16). In this study, the discovery of mutations in cell cycle genes *CDK6* and its homolog *CDK4* in both the BA and IMA regions was particularly noteworthy, with mutation abundance being higher than that of *KRAS*, reaching up to 49.01% and 51.45%. *CDK6* specifically regulates the transcription of tumor-related genes such as vascular endothelial growth factor-A (*VEGF-A*) and early growth response gene-1 (*EGR1*) (7, 8), thereby promoting the transcription of genes from G1 to S phase. Yu and colleagues (17) identified *CDK6* as one of the mRNAs differentially expressed in lung adenocarcinoma (LUAD) and lung squamous cell carcinoma (LUSC) through mRNA and circRNA expression patterns comparison. Their analysis of expression profile data and clinical information of the two subtypes from TCGA concluded that overexpression of *CDK6* predicted a poor prognosis in LUAD. Induction of *CDK6* by tripartite motif-containing 59 (*TRIM59*) contributes to the epithelial-to-mesenchymal transition (EMT) process, while *CDK4/6* mutations are associated with a poor prognosis in *KRAS*-mutant non-small-cell lung carcinoma (NSCLC), highlighting the role of *CDK4/6* mutations as oncogenic factors that promote tumor growth and metastasis (17, 18). Importantly, in this case, the variant allele frequency (VAF) of *CDK6* was higher in the IMA region than in the BA, further supporting the evidence of malignant transformation of the BA into IMA.

## Conclusions

In summary, we report a rare case of malignant transformation of BA into IMA with identified mutations in *KRAS* and *CDK6*. This case provides histological and genetic evidence supporting the potential for malignant transformation of BA into IMA, highlighting KRAS and CDK6 as possible driver genes in this process. These findings contribute to a better understanding of the biological behavior of BA and emphasize the importance of regular imaging follow-up for patients. Furthermore, this case underscores the need for additional studies to improve the understanding of the pathological features, genetic characteristics, and prognosis of BA.

## Data Availability

The original contributions presented in the study are included in the article/supplementary material. Further inquiries can be directed to the corresponding authors.
